# Optimization of Gas-Liquid Sulfonation in Cross-Shaped Microchannels for α-Olefin Sulfonate Synthesis

**DOI:** 10.3390/mi16060638

**Published:** 2025-05-28

**Authors:** Yao Li, Yingxin Mu, Muxuan Qin, Wei Zhang, Wenjin Zhou

**Affiliations:** Shanxi Key Laboratory of Chemical Product Engineering, College of Chemistry and Chemical Engineering, Taiyuan University of Technology, Taiyuan 030024, China; 19834520262@163.com (Y.L.); 15340708242@163.com (Y.M.); qinmuxuan01@163.com (M.Q.); zhouwenjin@tyut.edu.cn (W.Z.)

**Keywords:** microchannel reactor, α-olefin sulfonate, gas-liquid sulfonation, process intensification, multi-objective optimization

## Abstract

The gas-liquid sulfonation of α-olefin sulfonate (AOS) in falling film reactors faces significant limitations, primarily due to poor mass transfer efficiency and excessive byproduct formation. To overcome these challenges, a novel cross-shaped microchannel reactor was developed for the continuous gas-liquid sulfonation of α-olefin (AO) with gaseous sulfur trioxide (SO_3_). The influence of key process parameters, including gas-phase flow rate, reaction temperature, SO_3_/AO molar ratio, and SO_3_ volume fraction, on product characteristics and their interactions was systematically investigated using the single-factor experiment and response surface methodology (RSM). A high-precision empirical model (coefficient of determination, *R*^2^ = 0.9882) to predict product content was successfully constructed. To achieve multi-objective optimization considering product active substance content and energy efficiency, a strategy combining a two-population genetic algorithm with the entropy-weighted TOPSIS (Technique for Order of Preference by Similarity to Ideal Solution) method was implemented. Optimal conditions were determined as follows: gas-phase flow rate of 228 mL/min, reaction temperature of 52 °C, SO_3_/AO molar ratio of 1.27, and SO_3_ volume fraction of 4%. Compared to conditions optimized solely by RSM, this multi-objective approach achieved a significant 10% reduction in energy efficiency, with only a marginal 3.8% decrease in active substance content. This study demonstrates the feasibility and advantages of microreactors for the efficient and green synthesis of AOS.

## 1. Introduction

α-Olefin sulfonates (AOS), a prominent class of anionic surfactants, have garnered significant attention for their widespread application in domestic detergents and diverse industrial processes [1,2]. This is attributed to their advantageous properties, including excellent hard water tolerance, ready biodegradability, effective wetting capabilities, and a mild, low-irritation profile to human skin [3,4,5]. Consequently, AOS has emerged as a promising alternative to conventional surfactants such as alkylbenzene sulfonates and fatty alcohol ether sulfates, potentially mitigating some of their associated environmental and health concerns [6,7].

Conventional reactor designs, such as kettle and falling-film reactors, employed in industrial sulfonation processes, have a specific surface area that is only 1/10–1/1000 that of a microreactor [8], and often encounter challenges associated with inefficient mixing and inadequate heat removal [9,10]. The rapid, highly exothermic nature of these reactions can lead to non-uniform temperature profiles, promoting undesirable side reactions that negatively impact product quality and diminish equipment lifespan [11,12]. Microchannel reactors, recognized as powerful process intensification tools, offer a compelling alternative for such strong exothermic reactions [13,14]. Their characteristic micro-scale dimensions facilitate dramatically enhanced heat and mass transfer rates compared to conventional reactors [15,16]. This not only accelerates reaction kinetics but also enables precise control over reaction conditions, effectively suppressing side reactions and improving product selectivity [17,18].

The potential of microchannel reactors for the synthesis of sulfonate anionic surfactants has been demonstrated in several recent studies [19,20,21,22]. Xu et al. [19] utilized a continuous stirred kettle microreactor for the synthesis of cetylbenzenesulfonic acid. Geng et al. [20] implemented a continuous flow sulfonation process in a microreactor to optimize the conventional sulfonation of dodecylbenzene with dilute SO_3_. Furthermore, Yuan et al. [21] successfully synthesized dodecylbenzenesulfonic acid using a helicoidal tube microreactor, while Xu et al. [22] employed a specific microstructured microchip for the efficient preparation of fatty acid methyl ester sulfonates. These studies highlight the versatility and efficacy of microreactor technology for sulfonation reactions.

While microchannel reactors have shown promise for AOS synthesis, previous studies, such as the work by Ma et al. [23] on the sulfonation of 1-tetradecene with liquid SO_3_ in a microchannel and the multistep cascade synthesis investigated by Xu et al. [24], have primarily focused on liquid-liquid homogeneous sulfonation systems. These systems necessitate dissolving SO_3_ in organic solvents like dichloroethane, introducing additional separation and purification costs, as well as posing environmental and health hazards [25,26]. These drawbacks are incongruous with the green chemistry principles driving modern chemical manufacturing. Utilizing gaseous SO_3_ as the sulfonating agent obviates the need for subsequent solvent separation; however, it introduces the complexity of managing gas-liquid two-phase flow within the microchannel, which significantly influences interfacial mass transfer efficiency [27,28]. Moreover, gas-liquid sulfonation reactions are inherently more vigorous than their liquid-liquid counterparts, posing a substantial challenge in effectively dissipating the large heat release and mitigating side reactions [21]. This efficient heat management is crucial for achieving process stability and control. To date, a systematic investigation of AOS synthesis via gas-liquid sulfonation remains unexplored.

Furthermore, while maximizing the active substance content is desirable, the associated energy costs must be carefully considered [29,30]. Therefore, global process optimization, balancing product quality with energy consumption, is essential for minimizing costs and maximizing overall efficiency [31]. Multi-objective optimization algorithms offer a powerful approach for navigating such complex design spaces [32,33]. For instance, Alexandrova et al. [34] successfully employed the NSGA-II algorithm to optimize polycyclic aromatic hydrocarbon hydrogenation temperatures, maximizing both cycloalkane yield and feedstock conversion. Similarly, Verhellen et al. [35] leveraged NSGA-II for efficient molecular design in small-molecule drug discovery, demonstrating its superiority over single-objective methods. Consequently, applying these advanced optimization strategies to simultaneously enhance product quality and minimize energy consumption is a critical aspect in developing a sustainable and economically viable continuous AOS synthesis process via gas-liquid microchannel sulfonation.

This study addresses the critical challenges associated with conventional AOS synthesis via SO_3_ gas-liquid sulfonation, particularly the difficulties in controlling the highly exothermic reaction and mitigating side reactions. We report, for the first time, the development and systematic optimization of a continuous-flow AOS synthesis process employing gas-liquid sulfonation within a cross-shaped microchannel reactor. The influence of key process parameters, including gas-phase flow rate, SO_3_ concentration, reactant molar ratio, reaction temperature, and residence time, on AOS yield and selectivity within the microchannel was investigated in detail. Furthermore, to achieve cost-effective production, an improved two-population genetic algorithm was implemented for multi-objective optimization, aiming to identify the optimal operating conditions that simultaneously maximize active substance content and minimize energy consumption. This study provides a theoretical foundation and technical framework for the continuous, efficient, safe, and sustainable production of AOS, paving the way for the green manufacturing of high-performance surfactants.

## 2. Materials and Methods

### 2.1. Chemicals

1-Dodecene (purity > 95.0 wt%) was purchased from Macklin Biochemical Co., Ltd., Shanghai, China. Sulfur dioxide (SO_2_, standard gas) was obtained from Anxuhongyun Science and Technology Development Co., Ltd., Taiyuan, China. Anhydrous ethanol (>99.7 wt%), isopropyl alcohol (>99.7 wt%), and petroleum ether (analytical grade) were supplied by Sinopharm Chemical Reagent Co., Ltd., Shanghai, China. Sodium hydroxide (>98.0 wt%) was purchased from the Kemiou Chemical Reagent Co., Ltd., Tianjin, China. Methanol (chromatographic grade, >99.9 wt%) was obtained from Aladdin Biochemical Technology Co., Ltd., Shanghai, China.

### 2.2. Synthesis of AOS in the Microchannel Reactor

**Figure 1:**
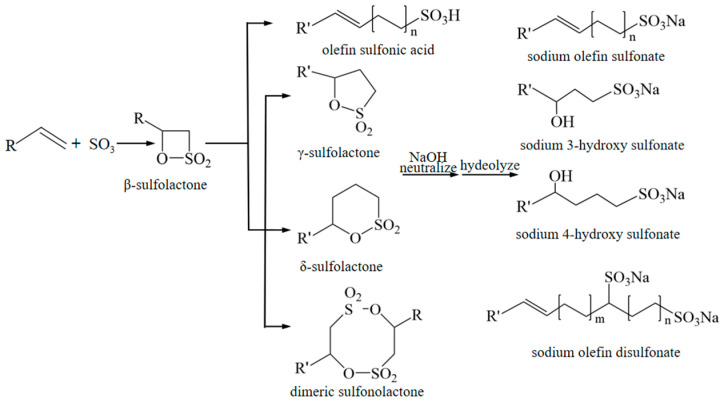
AOS synthesis mechanism.

AOS synthesis was conducted in a custom-built gas-liquid microsulfonation system featuring a cross-shaped microchannel chip. The proposed reaction mechanism, illustrated in Figure 1, involves a complex pathway with several intermediate and side reactions. Initially, SO_3_ undergoes electrophilic addition to the α-olefin, forming the unstable β-sultone intermediate. This intermediate can subsequently isomerize to various products, including olefin sulfonic acid, γ-sultone, δ-sultone, and dimeric sultones. Neutralization of the olefin sulfonic acid yields sodium olefin sulfonate. Under alkaline conditions, the sultones undergo hydrolysis and neutralization to form hydroxyalkanesulfonates, which can further convert to AOS at elevated temperatures.

**Figure 2:**
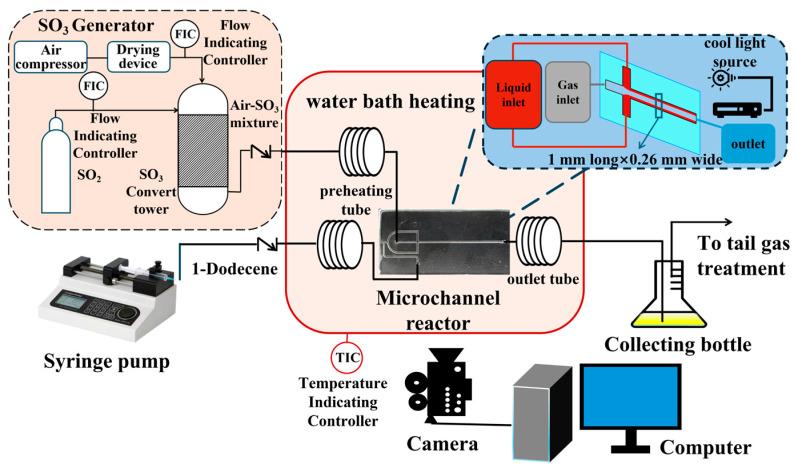
Gas-liquid microsulfonation reaction system.

**Figure 3:**
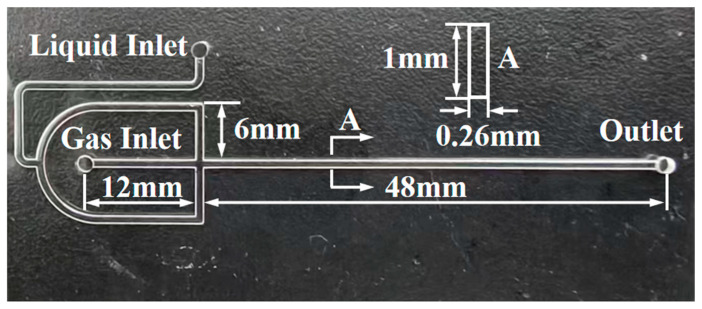
Cross-shaped microchannel chip.

The experimental setup, depicted in Figure 2, comprised an SO_3_ generator (FJEE-III, China Research Institute of Daily-use Chemical Industry) and a cross-shaped microchannel chip, depicted in Figure 3, which is fabricated from borosilicate glass through a glass etching process. The setup also included several PTFE tubes, a micro-syringe pump (LSP01-3A, Baoding Lange Constant Flow Pump Co., Ltd., Baoding, China), a constant-temperature water bath, a high-speed camera, an SO_3_ tail gas treatment unit, and a computer workstation. The system allowed precise control of the reaction temperature and gas-liquid flow rates. SO_3_ is generated by the catalytic conversion of SO_2_ with dry air in a reformer tower at a reaction temperature of 465 °C over a V_2_O_5_ catalyst, with the SO_3_ concentration and gas flow rate regulated by a mass flow controller. The liquid-phase reactant, 1-dodecene, was delivered via the micro-syringe pump and preheated to the reaction temperature within a PTFE tubing (1.6 mm O.D. × 0.6 mm I.D.).

**Figure 4:**
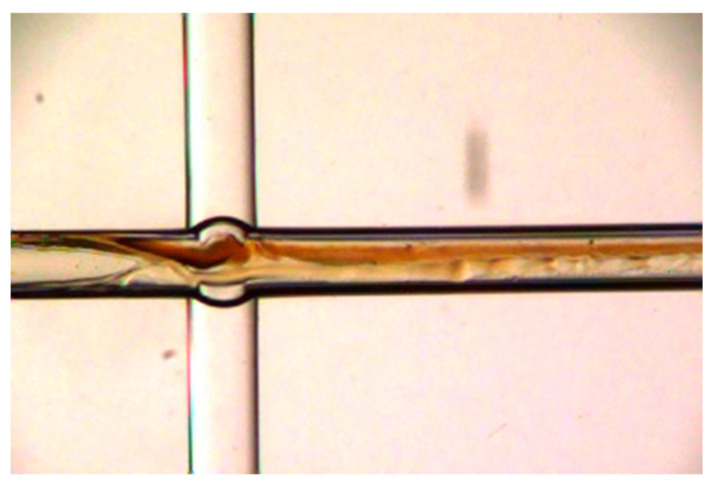
Gas-liquid annular flow in microchannel.

The gas and liquid streams were introduced into the cross-shaped microchannel—the gas centrally and the liquid from the opposing ends—forming an annular flow regime, as shown in Figure 4, with a gas core surrounded by a liquid film. The reaction mixture then flowed into downstream PTFE tubing (1.6 mm O.D. × 0.6 mm I.D.) for further reaction; the length of the tubing was 2 m. This configuration, within the microchannel in chip and PTFE tubing, facilitated efficient mixing and rapid reaction. The product stream was collected, while the remaining gas flowed into the SO_3_ tail gas treatment unit. The preheating tubing, microchannel chip, and outlet tubing were immersed in a constant-temperature water bath. The sulfonation products were neutralized, hydrolyzed, and subsequently analyzed by liquid chromatography.

### 2.3. Product Analysis Methods

The active substance content of the sulfonated products was determined by high-performance liquid chromatography, (HPLC, LC-16, Shimadzu Co., Ltd., Suzhou, China) equipped with a differential refractive index detector. The chromatographic conditions are summarized in Table 1.

An external standard method was employed for quantification. A calibration curve was constructed by analyzing a series of AOS standard solutions of known concentrations under the specified chromatographic conditions (Table 1) and plotting peak areas against concentration. Sulfonated product samples were dissolved in methanol, filtered through a membrane filter, and analyzed by HPLC. The active substance content was calculated using Equation (1):

(1) $$Y_{AOS}=\frac{C_{AOS}\times V\times D_{t}}{m}\times 100\%$$

where *Y_AOS_* is the content of the active substance, %; *C_AOS_* is the concentration of AOS in the sample, g/mL; *V* is the volume of the sample constant volume, mL; *D_t_* is the dilution times; and *m* is the sampling mass of sulfonated product, g.

### 2.4. Box-Behnken Response Surface Experimental Design

To systematically investigate the influence of key operating parameters (gas-phase flow rate, reaction temperature, SO_3_/α-olefin molar ratio, and SO_3_ volume fraction) on the sulfonation of 1-dodecene with gaseous SO_3_ in a cross-shaped microreactor, response surface methodology (RSM) coupled with a Box-Behnken design (BBD) was employed. This approach facilitated the analysis of factor interactions and the determination of optimal operating conditions through multiple regression analysis. A four-factor, three-level BBD was implemented. The ranges for each factor, initially determined through single-factor experiments, are presented in Table 2.

## 3. Results and Discussion

### 3.1. Single-Factor Experimental Analysis

**Figure 5:**
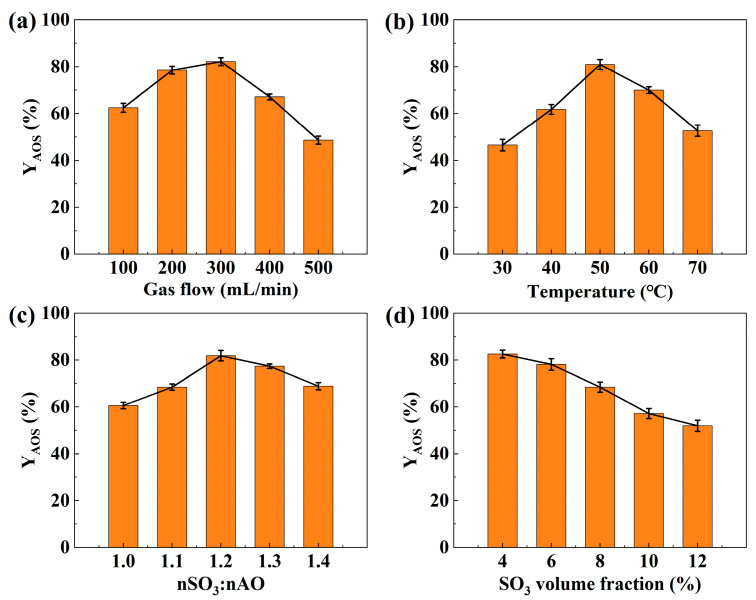
Effect of various factors on the activity content: (**a**) effect of gas phase flow rate; (**b**) effect of reaction temperature; (**c**) effect of SO_3_/AO molar ratio; (**d**) effect of SO_3_ volume fraction.

The influence of gas-phase flow rate, reaction temperature, SO_3_/α-olefin (AO) molar ratio, and SO_3_ volume fraction on the active substance content was initially investigated through single-factor experiments. The results are presented in Figure 5.

#### 3.1.1. Effect of Gas-Phase Flow Rate

The SO_3_-air mixture flow rate is crucial for controlling the flow regime and mixing efficiency in gas-liquid sulfonation, the influence of this flow rate is particularly pronounced in microchannels. As this is a heterogeneous gas-liquid reaction, mixing directly impacts the reaction process. The effect of gas flow rate was investigated at a temperature of 50 °C, an SO_3_/AO molar ratio of 1.2, and an SO_3_ volume fraction of 4%. Increasing the gas flow rate from 100 mL/min to 300 mL/min enhanced the active substance content. This increase likely promoted turbulence within the annular flow, enlarging the gas-liquid interfacial area and thus improving mass transfer of SO_3_ from the gas to the liquid phase. However, further increasing the gas flow rate from 300 mL/min to 500 mL/min led to a decrease in active substance content. At higher gas velocities, increased fluctuations can destabilize the annular flow, potentially disrupting the liquid film and reducing mass transfer efficiency. Furthermore, higher flow rates shorten the residence time, limiting the residence time between SO_3_ and the α-olefin and promoting the escape of unreacted SO_3_.

#### 3.1.2. Effect of Reaction Temperature

The sulfonation reaction is highly exothermic, with its rate strongly dependent on temperature. The reaction temperature was controlled using a constant-temperature water bath. The influence of temperature on active substance content was investigated at a gas flow rate of 300 mL/min, an SO_3_/AO molar ratio of 1.2, and an SO_3_ volume fraction of 4%. Increasing the temperature from 30 °C to 50 °C significantly enhanced the active substance content. This is consistent with the Arrhenius equation, where higher temperatures increase the reaction rate constant and accelerate the reaction, leading to higher α-olefin conversion. Additionally, the decreased liquid viscosity at elevated temperatures likely enhances SO_3_ diffusion within the liquid phase, further promoting the reaction. However, further temperature increases to 60 °C and 70 °C resulted in a significant decline in active substance content. This suggests that at higher temperatures, side reactions become more prominent, the byproducts primarily consist of disulfonates from oversulfonation and unconverted sulfonolactones, leading to increased byproduct formation and consequently reducing the active substance content. Therefore, an optimal temperature of approximately 50 °C is indicated for maximizing active substance content.

#### 3.1.3. Effect of SO_3_/AO Molar Ratio

Stoichiometrically, the reaction between SO_3_ and AO requires a 1:1 molar ratio. However, due to mass transfer limitations and competing side reactions, excess SO_3_ is typically required to achieve optimal conversion. The SO_3_/AO molar ratio was controlled by adjusting the liquid flow rate. Its effect on the active substance content was investigated at a gas flow rate of 300 mL/min, a temperature of 50 °C, and an SO_3_ volume fraction of 4%. Increasing the molar ratio from 1.0 to 1.2 significantly enhanced the active substance content, reaching a maximum value. This increase can be attributed to the reduced liquid film thickness at lower liquid flow rates (corresponding to higher molar ratios), which shortens the gas-liquid mass transfer distance and facilitates SO_3_ transfer into the liquid phase. The resulting higher SO_3_ concentration in the liquid phase promotes more complete α-olefin conversion. However, further increases in the molar ratio to 1.3 and 1.4 led to a decline in active substance content. This suggests that excessive SO_3_ can lead to over-sulfonation and enhanced side reactions, increasing byproduct formation. Furthermore, the reduced liquid flow rate at higher molar ratios may destabilize the annular flow, resulting in non-uniform α-olefin distribution within the microchannel and hindering the reaction.

#### 3.1.4. Effect of SO_3_ Volume Fraction

The mixture of SO_3_ and air was used as the sulfonating agent, with the SO_3_ volume fraction representing the sulfonating agent concentration. The effect of SO_3_ volume fraction on active substance content was investigated at a gas flow rate of 300 mL/min, a temperature of 50 °C, and an SO_3_/AO molar ratio of 1.2. Contrary to initial expectations, the active substance content decreased as the SO_3_ volume fraction increased from 4% to 12%. While a higher SO_3_ volume fraction should theoretically enhance the mass transfer driving force and reaction rate, maintaining a constant molar ratio at higher SO_3_ concentrations necessitates a corresponding increase in the liquid flow rate. This decrease in the gas-to-liquid flow ratio thickens the liquid film in the annular flow regime, increasing the liquid-phase mass transfer resistance. Consequently, SO_3_ accumulates at the gas-liquid interface, leading to a localized high-temperature zone due to the rapid exothermic reaction. This localized heating further promotes side reactions. Additionally, product accumulation at the interface hinders mass transfer, ultimately reducing the active substance content. These results indicate that an SO_3_ volume fraction of 4% provides optimal mass transfer efficiency under the investigated conditions.

### 3.2. BBD Response Surface Experiment Analysis

Optimization and analysis of AOS synthesis were performed using response surface methodology (RSM) coupled with a Box-Behnken design (BBD). The effects of gas flow rate, temperature, SO_3_/AO molar ratio, and SO_3_ volume fraction on the active substance content of AOS were investigated to determine the optimal process conditions. A four-factor, three-level BBD was employed. Table 3 presents the factors and their corresponding levels, which were determined based on preliminary single-factor experiments.

#### 3.2.1. Model Construction and Fitting

Design-Expert software (Version 12, Stat-Ease Inc., Minneapolis, MN, USA) was used for the experimental design, model prediction, and statistical analysis. A four-factor, three-level BBD, comprising 29 randomized experiments that included five center point replicates for pure error estimation, was employed. Table 4 presents the coded factor levels, experimental active substance content, and predicted active substance content for each run. A quadratic polynomial regression model (Equation (2)) was developed to correlate the response with the independent variables:

(2) $$\begin{array}{l} Y=80.39-4.96A+2.06B+2.08C-4.42D+0.3125AB-1.05AC+2.38AD\\ +1.00BC+0.0625BD-2.09CD-8.38A^{2}-7.11B^{2}-5.55C^{2}-2.64D^{2} \end{array}$$

where *Y* represents the active substance content of the product; *A*, *B*, *C*, and *D* represent the gas flow rate, temperature, SO_3_/AO molar ratio, and SO_3_ volume fraction, respectively; *AB*, *AC*, *AD*, *BC*, *BD*, and *CD* represent the interaction terms between the independent variables; and *A*^2^, *B*^2^, *C*^2^, and *D*^2^ represent the quadratic terms of the respective independent variables.

The significance of the quadratic regression model was evaluated using analysis of variance (ANOVA). The ANOVA results for optimizing active substance content are presented in Table 5. A large *F*-value indicates high statistical significance, while a *p*-value less than 0.05 indicates statistical significance. As shown in Table 5, the model *F*-value of 113.67 (*p* < 0.0001) confirms the significance of the model. The terms *A* (gas flow rate), B (temperature), *C* (SO_3_/AO molar ratio), *D* (SO_3_ volume fraction), *AD*, *CD*, *A*^2^, *B*^2^, *C*^2^, and *D*^2^ were statistically significant. The magnitude of the *F*-value for each term reflects its influence on active substance content, with the order of significance for individual factors being gas flow rate > SO_3_ volume fraction > temperature > SO_3_/AO molar ratio. The lack-of-fit *F*-value of 2.25 (*p* = 0.2263) indicates that the lack of fitness is not significant relative to pure error. The low *p*-value and small pure error suggest excellent repeatability of the experimental data.

The model’s goodness of fit was assessed using the coefficient of determination (*R*^2^) and the adjusted *R*^2^ (*R*^2^*_ad__j_*). As shown in Table 6, the *R*^2^ value of 0.9882 indicates a strong correlation between the independent variables and the response. The *R*^2^*_ad_*_j_ value of 0.9763 further supports the model’s adequacy, suggesting a good fit between the regression equation and the experimental data. The predicted *R*^2^*_pre_* (0.9415) is in reasonable agreement with the adjusted *R*^2^, with a difference of less than 0.2. The low coefficient of variation (CV) of 1.57% (<10%) indicates good model reproducibility. Furthermore, the adequate precision value (signal-to-noise ratio) of 29.9645 (>4) confirms the model’s adequacy for navigating the design space. Diagnostic plots, including predicted vs. actual values, normal probability plots, and externally studentized residuals, were examined to verify the model’s statistical adequacy.

**Figure 6:**
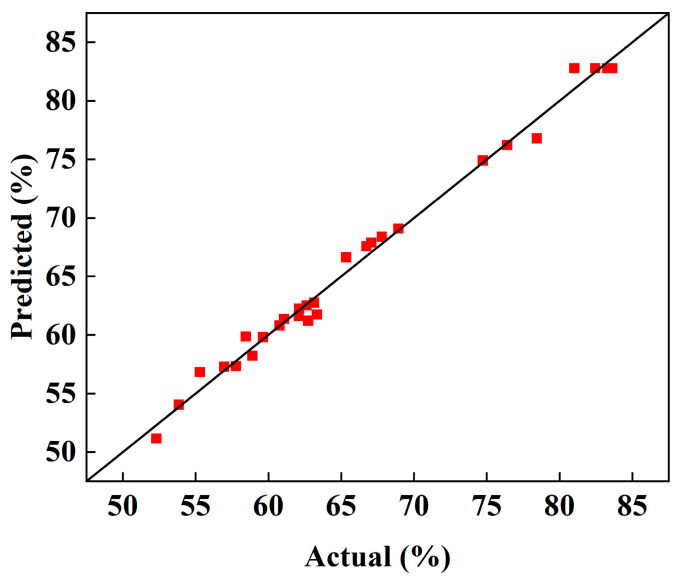
Distribution of predicted and actual values.

**Figure 7:**
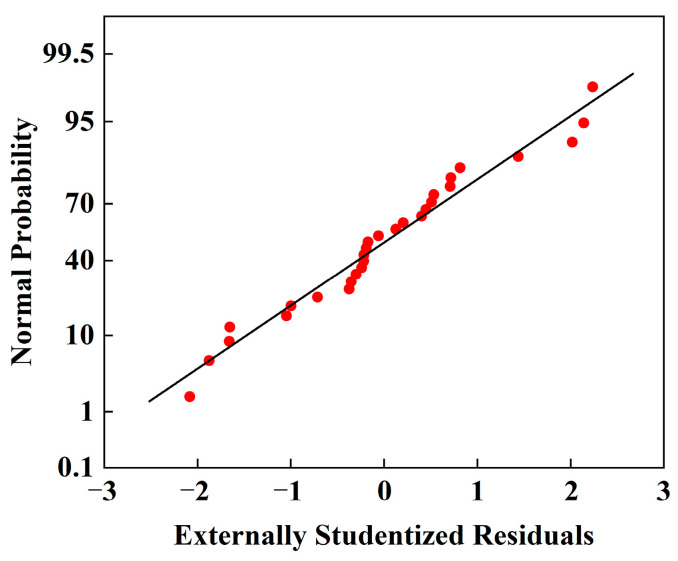
Normal probability distribution of residuals.

The model’s statistical adequacy was assessed using diagnostic plots, including predicted vs. actual values (Figure 6), normal probability plots, and externally studentized residuals (Figure 7). As shown in Figure 6, the predicted and actual values are in good agreement. The normal probability plot (Figure 7) and the distribution of externally studentized residuals (within ±3) further confirm the model’s accuracy and validate the normality assumptions.

#### 3.2.2. Response Surface Analysis

**Figure 8:**
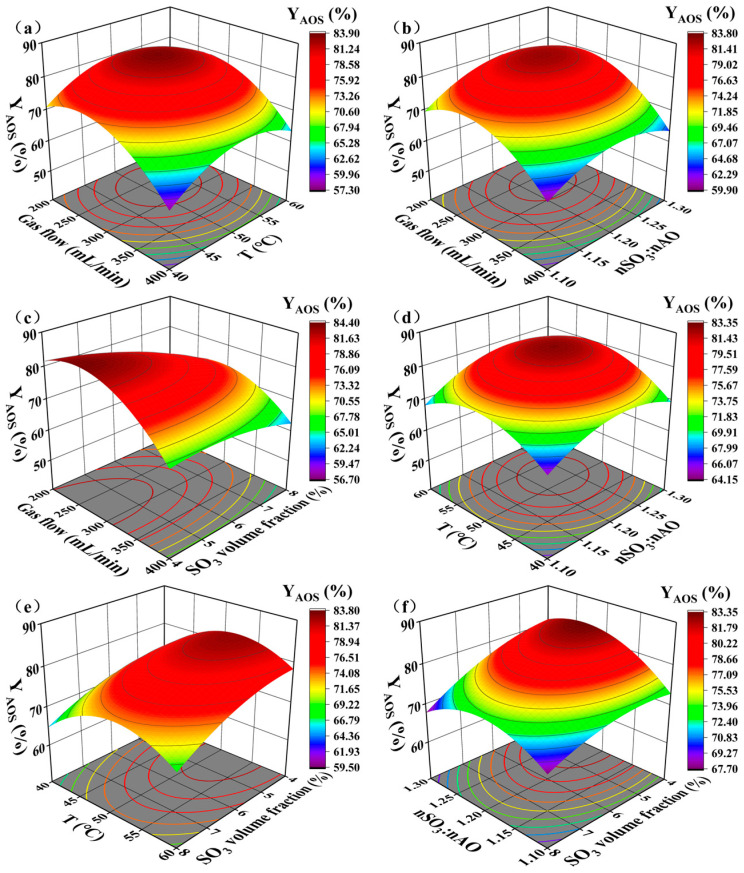
The response surface plots illustrating the interactive effects of the process parameters on AOS synthesis in the microchannel reactor: (**a**) gas flow rate and temperature; (**b**) gas flow rate and SO_3_/AO molar ratio; (**c**) gas flow rate and SO_3_ volume fraction; (**d**) temperature and SO_3_/AO molar ratio; (**e**) temperature and SO_3_ volume fraction; and (**f**) SO_3_ volume fraction and SO_3_/AO molar ratio.

Three-dimensional (3D) response surface plots and their corresponding contour plots (Figure 8) were used to visualize the interactive effects of the independent variables on the active substance content of AOS. These plots provide insights into the significance of the interactions and their influence on AOS synthesis.

Figure 8a,b illustrates the interactions between gas flow rate and temperature and gas flow rate and SO_3_/AO molar ratio, respectively. In both cases, the active substance content initially increases and then decreases, forming a peak in the central region of the response surface. The elliptical contours, with their long axes oriented along the gas flow rate axis, suggest that gas flow rate has a more significant influence than temperature or molar ratio, and that the interactions between these factors are relatively weak. Figure 8c depicts the interaction between gas flow rate and SO_3_ volume fraction. The irregular shape of the response surface, with one diagonally rising sharply and the other falling, indicates a significant interaction between these two factors. At low SO_3_ volume fractions, the active substance content is highly sensitive to changes in gas flow rate, initially increasing and then decreasing significantly. This suggests that the gas flow rate is the dominant factor under these conditions. At higher SO_3_ volume fractions, the active substance content still exhibits an initial increase followed by a decrease with increasing gas flow rate, indicating that optimizing gas flow conditions can improve mass transfer even at suboptimal SO_3_ concentrations. However, low SO_3_ volume fractions are generally favored for achieving high active substance content. Optimizing the gas flow rate under these low SO_3_ volume fraction conditions maximizes the synergistic effect of these two parameters, leading to enhanced mass transfer and higher active substance content.

Figure 8d shows the interaction between temperature and SO_3_/AO molar ratio. The uniform slopes of the response surface and circular contours indicate a negligible interaction between these factors. Figure 8e,f illustrates the interactions of SO_3_ volume fraction with temperature and molar ratio, respectively. The stable trend of the temperature response surface with decreasing SO_3_ volume fraction (Figure 8e) suggests a weak interaction. In contrast, the sloped response surface and tilted elliptical contours in Figure 8f reveal a significant interaction between SO_3_ volume fraction and molar ratio. At a fixed SO_3_ volume fraction of 4%, the active substance content increases sharply as the molar ratio increases from 1.1 to 1.2, representing the steepest region of the response surface. This highlights the synergistic effect of these two factors in maximizing active substance content. However, further increasing the molar ratio from 1.2 to 1.3 leads to a decrease in active substance content, likely due to the increasing dominance of side reactions, which are not suppressed by the low SO_3_ volume fraction. At an SO_3_ volume fraction of 8%, the active substance content still exhibits an initial increase followed by a decrease with an increasing molar ratio, but the overall active substance content is lower, suggesting that the higher SO_3_ concentration limits the achievable active substance content.

#### 3.2.3. Experimental Validation of Optimized Conditions

The validity of the RSM model was assessed by comparing experimental results with model predictions under optimized conditions. The optimized conditions for maximizing active substance content, determined using the model, are presented in Table 7, along with the corresponding predicted values.

To minimize experimental errors, triplicate experiments were conducted under the optimized conditions. The average experimental active substance content was 84.45%, which closely matched the predicted value of 85.14%. This strong agreement validates the RSM model and confirms the reliability of the optimized sulfonation conditions for α-olefins in the cross-shaped microreactor.

While the RSM optimization above focused solely on maximizing active substance content, it did not consider power consumption. To develop a more efficient, sustainable, and economical AOS process, a multi-objective optimization approach is necessary. This approach aims to maximize active substance content while simultaneously minimizing power consumption. However, these objectives are often conflicting, as increasing active substance content typically requires higher power input. Therefore, a multi-objective optimization algorithm is employed to navigate this trade-off and identify the Pareto optimal solutions.

### 3.3. Multi-Objective Optimization of Process Parameters Using NSGA-II and Entropy-Weighted TOPSIS

#### 3.3.1. Multi-Objective Optimization of AOS Synthesis Using a Dual-Population NSGA-II and Entropy-Weighted TOPSIS

**Figure 9:**
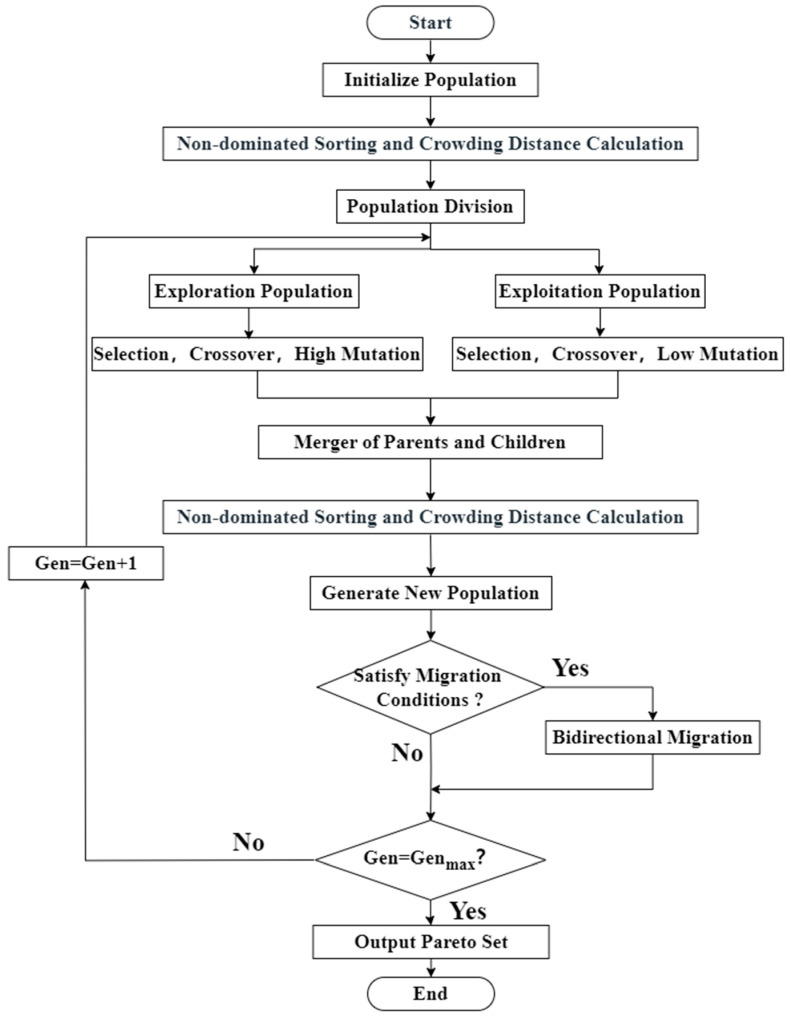
Flowchart of the improved NSGA-II algorithm.

The non-dominated genetic algorithm II (NSGA-II) is a widely used multi-objective optimization algorithm known for its robustness and efficiency in generating Pareto optimal solutions [36,37]. Its core mechanisms include non-dominated sorting based on Pareto dominance and crowding distance calculation to maintain diversity within the Pareto front. To enhance global search capability and mitigate premature convergence, a dual-population strategy was incorporated into the standard NSGA-II algorithm. This strategy employs two independent sub-populations evolving in parallel, with periodic exchange of a small number of individuals to promote diversity and explore the search space more effectively. This improved NSGA-II algorithm was used to optimize both active substance content and power consumption, identifying reaction conditions that balance product quality and energy efficiency. This approach offers valuable guidance for industrial production. The structure of the improved NSGA-II algorithm is illustrated in Figure 9.

The entropy-weighted TOPSIS method is a multi-criteria decision-making approach that combines the objectivity of entropy weighting with the relative performance evaluation of TOPSIS [38]. Entropy weighting determines the weights of different criteria based on the variability of the corresponding data, thereby minimizing subjective bias. The TOPSIS method calculates the relative closeness of each solution in the Pareto front to the ideal solution. The solution with the highest relative closeness is then selected as the optimal solution.

Entropy weighting was employed to determine the weights of each objective function. First, the Pareto optimal solution set matrix *X* = (*x_ij_*)*_m×n_*, where *m* is the number of solutions and *n* is the number of objectives, was normalized. Equations (3) and (4) were used to normalize the benefit-type (maximization) and cost-type (minimization) indicators, respectively, resulting in the normalized matrix *R* = (*r_ij_*)*_m×n_*:

(3) $$r_{ij}=\frac{x_{ij}-\min(x_{j})}{\max(x_{j})-\min(x_{j})}$$

(4) $$r_{ij}=\frac{\max(x_{j})-x_{ij}}{\max(x_{j})-\min(x_{j})}$$

Next, the information entropy and entropy weights were calculated. Equation (5) was used to transform the normalized values *r_ij_* into probabilities *p_ij_*. The information entropy *e_j_* and entropy weight *w_j_* for the *j*th objective were then calculated using Equations (6) and (7), respectively:

(5) $$p_{ij}=\frac{r_{ij}}{\sum_{k=1}^{m}r_{kj}}$$

(6) $$e_{j}=-\frac{1}{\ln m}\sum_{i=1}^{m}p_{ij}\ln p_{ij}$$

(7) $$w_{j}=\frac{1-e_{j}}{\sum_{k=1}^{n}\left(1-e_{k}\right)}$$

The information entropy reflects the degree of variability or information content within the data for each indicator. A larger entropy weight indicates a greater influence of the corresponding objective in the overall evaluation. Finally, the normalized probabilities *p_ij_* were weighed by the corresponding entropy weights *w_j_* to construct the weighted decision matrix *v_ij_* according to Equation (8). This weighted matrix, incorporating both the indicator values and their relative importance, serves as the basis for the subsequent TOPSIS analysis.

(8) $$v_{ij}=w_{j}\cdot p_{ij}$$

The first step in the TOPSIS analysis involves determining the positive ideal solution (PIS) and negative ideal solution (NIS). The PIS, denoted as *V_j_^+^*, is composed of the maximum values (max(*v_ij_*)) for each indicator, representing the ideal scenario where all objectives are maximized. Conversely, the NIS, denoted as *V_j_^−^*, consists of the minimum values (min(*v_ij_*)) for each indicator, representing the worst-case scenario.

Next, the Euclidean distances between each solution and the PIS (*D_i_^+^*) and NIS (*D_i_^−^*) were calculated using Equations (9) and (10). These distances represent the proximity of each solution to the ideal and worst-case scenarios, respectively.

(9) $$D_{i}^{+}=\sqrt{\sum_{j=1}^{n}{\left(v_{ij}-V_{j}^{+}\right)}^{2}}$$

(10) $$D_{i}^{-}=\sqrt{\sum_{j=1}^{n}{\left(v_{ij}-V_{j}^{-}\right)}^{2}}$$

Finally, the relative closeness (*C_i_*) of each solution to the PIS was calculated using Equation (11):

(11) $$C_{i}=\frac{D_{i}^{-}}{D_{i}^{+}+D_{i}^{-}}$$

A higher relative closeness (*C_i_*) indicates that the solution is closer to the PIS and farther from the NIS, representing a more desirable performance. The solutions were ranked based on their *C_i_* values, allowing for the selection of the optimal solution.

#### 3.3.2. Objective Function

This study aimed to optimize both active substance content and power consumption in gas-liquid microsulfonation using a multi-objective optimization approach. Two objective functions were defined, representing active substance content and power consumption, respectively. The active substance content objective function (Equation (12)) was derived from the response surface model, using the actual values (rather than coded values) of the independent variables: gas flow rate (*A*), temperature (*B*), SO_3_/AO molar ratio (*C*), and SO_3_ volume fraction (*D*).

(12) $$\begin{array}{l} Y=-1043.90292+0.497270A+6.004B+1417.0633C+15.20767D\\ +0.000313AB-0.105AC+0.011888AD+1.0025BC-0.021875BD\\ -10.45000CD-0.000846A^{2}-0.069466B^{2}-563.40833C^{2}-0.619458D^{2} \end{array}$$

Power consumption during the sulfonation process primarily arises from the pressure drop associated with material transport and the electrical energy consumed by the pumps for water bath heating and reactant delivery. Therefore, the power consumption model described by Equation (13) was employed:

(13) $$P=\frac{\Delta p_{1}Q_{g}}{\eta_{1}}+\frac{\Delta p_{2}Q_{l}}{\eta_{2}}+kS\Delta T+P_{g}+P_{l}$$

where *P* is the total power consumption (kW), Δ*p* is the gas-liquid pressure drop (determined from the pressure difference between the inlet and outlet piping), *Q_g_* and *Q_l_* are the gas and liquid flow rates, respectively, *η*_1_ and *η*_2_ are the efficiencies of the air compressor and syringe pump, respectively, *k* is the heat transfer coefficient of the water bath, *S* is the heat transfer area (the power consumed for water bath heating is assumed equal to the heat loss to the surroundings), and *P_g_* and *P_l_* are the base power consumption of the air compressor and syringe pump, respectively.

The physical parameters in Equation (13) were expressed in terms of the independent variables from the response surface model (A, B, C, and D) using the experimental conditions and equipment specifications. Substituting these relationships along with the measured pressure drops and temperature differences into Equation (13) yielded the power consumption model in terms of A, B, C, and D (Equation (14)).

(14) $$X=0.00718A+0.00177\frac{AD}{C}+0.00213(B-25)+1.5$$

Based on the response surface experiments, the following ranges were defined for the independent variables: gas flow rate (*A*) 200–400 mL/min, reaction temperature (*B*) 40–60 °C, SO_3_/AO molar ratio (*C*) 1.1–1.3, and SO_3_ volume fraction (*D*) 4–8%. A bi-objective optimization was performed, aiming to maximize active substance content (*Y*) and minimize power consumption (*X*).

#### 3.3.3. Optimization and Decision Results

**Figure 10:**
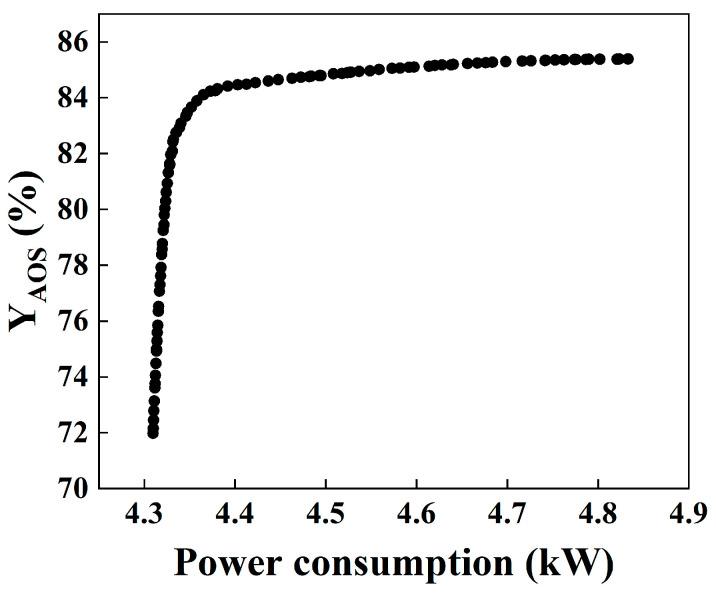
Pareto solution set for power consumption and active substance content.

The NSGA-II algorithm was implemented with the following parameters: crossover probability = 0.8, mutation probability (population 1) = 0.5, mutation probability (population 2) = 0.1, population size = 100, and number of generations = 200. The resulting Pareto front is shown in Figure 10. The optimal solution, identified from the Pareto front using the entropy-weighted TOPSIS method, is presented in Table 8.

Table 9 compares the optimal solution obtained from the multi-objective optimization (MOO) with the solution from the response surface methodology (RSM) maximizing active substance content. While the MOO solution resulted in a 3.8% decrease in active substance content compared to the RSM solution, it achieved a 10% reduction in power consumption. This demonstrates the effectiveness and feasibility of the multi-objective optimization approach in balancing active substance content and energy efficiency.

## 4. Conclusions

This study demonstrated the continuous-flow synthesis of α-olefin sulfonates (AOS) via gas-liquid sulfonation of SO_3_ and α-olefins (AO) in a cross-shaped microreactor. The rapid, highly exothermic reaction was systematically investigated and optimized. The results confirm that the cross-shaped microreactor provides excellent mass and heat transfer characteristics, significantly enhancing reaction efficiency and enabling the green and efficient synthesis of AOS.

A Box-Behnken design coupled with response surface methodology (RSM) was employed to investigate the effects of four key process parameters—gas flow rate, temperature, SO_3_/AO molar ratio, and SO_3_ volume fraction—on AOS active substance content. The order of significance of these factors was determined to be: gas flow rate > SO_3_ volume fraction > temperature > SO_3_/AO molar ratio. Significant interactions were observed between gas flow rate and SO_3_ volume fraction, and between SO_3_ volume fraction and molar ratio. The resulting quadratic regression model accurately predicted AOS active substance content (R^2^ > 0.94), with its validity and goodness of fit rigorously confirmed by ANOVA.

To improve process sustainability and economic viability, a power consumption model for the gas-liquid microsulfonation system was developed. Using this model and the previously established active substance content prediction model, a multi-objective optimization was performed using an improved dual-population NSGA-II algorithm. This optimization aims to simultaneously maximize active substance content and minimize power consumption. The entropy-weighted TOPSIS method was then applied to select the optimal operating conditions from the resulting Pareto front. The optimal conditions were determined to be: gas flow rate = 226 mL/min, temperature = 52 °C, SO_3_/AO molar ratio = 1.27, and SO_3_ volume fraction = 4%. Under these conditions, an active substance content of 83% was achieved while simultaneously reducing power consumption by 10% compared to the conditions that solely maximize active substance content. This demonstrates the successful application of the multi-objective optimization approach in balancing product yield and energy efficiency.

This study successfully demonstrated a novel continuous-flow process for AOS synthesis in a microreactor. By integrating response surface methodology, a multi-objective optimization algorithm (improved dual-population NSGA-II), and an entropy-weighted TOPSIS decision-making method, optimal operating conditions were identified that balance high active substance content with minimized power consumption. This work provides a strong theoretical and experimental foundation for the development of more efficient, economical, and environmentally friendly AOS microreactor synthesis technologies. Furthermore, it highlights the significant potential of combining process intensification with intelligent optimization algorithms for addressing complex chemical process optimization challenges.

## Figures and Tables

**Table 1:** HPLC analytical conditions.

Chromatographic Conditions	Parameters
column	C_18_ column (250 × 4.6 × 5 μm)
mobile phase	methanol
detector	differential refractive index detector
flow rate (mL/min)	1
column temperature (°C)	40
feed volume (μL)	15

**Table 2:** Single-factor experimental variables and ranges.

Variables	Units	Ranges
gas-phase flow rate	mL/min	100, 200, 300, 400, 500
reaction temperature	°C	30, 40, 50, 60, 70
SO_3_/α-olefin molar ratio	—	1.0, 1.1, 1.2, 1.3, 1.4
SO_3_ volume fraction	%	4, 6, 8, 10, 12

**Table 3:** Ranges of experimental and coded values used in BDD design.

Variables	Units	Level		
		−1	0	1
gas-phase flow rate	mL/min	200	300	400
reaction temperature	°C	40	50	60
SO_3_/AO molar ratio	—	1.1	1.2	1.3
SO_3_ volume fraction	%	4	6	8

**Table 4:** Response surface experimental data.

No.	Factors				Response Value *Y* (%)	
	A	B	C	D	Experimental Value	Predicted Value
1	−1	−1	0	0	67.61	68.11
2	1	−1	0	0	58.4	57.57
3	−1	1	0	0	71.23	71.61
4	1	1	0	0	63.27	62.32
5	0	0	−1	−1	71.84	72.45
6	0	0	1	−1	80.9	80.79
7	0	0	−1	1	68.13	67.79
8	0	0	1	1	68.83	67.77
9	−1	0	0	−1	82.93	81.12
10	1	0	0	−1	66.57	66.46
11	−1	0	0	1	67.64	67.53
12	1	0	0	1	60.79	62.37
13	0	−1	−1	0	64.4	64.59
14	0	1	−1	0	68.23	66.71
15	0	−1	1	0	65.45	66.75
16	0	1	1	0	73.29	72.88
17	−1	0	−1	0	67.57	68.29
18	1	0	−1	0	60.13	60.48
19	−1	0	1	0	74.22	74.55
20	1	0	1	0	62.58	62.54
21	0	−1	0	−1	73.26	73.06
22	0	1	0	−1	77.43	77.06
23	0	−1	0	1	65.04	64.09
24	0	1	0	1	67.46	68.34
25	0	0	0	0	79.01	80.39
26	0	0	0	0	81.13	80.39
27	0	0	0	0	80.77	80.39
28	0	0	0	0	79.94	80.39
29	0	0	0	0	81.12	80.39

**Table 5:** Analysis of variance for response surface quadratic modeling.

Source	Square Sum	Degrees of Freedom	Mean Square	*F*-Value	*p*-Value	Significance
model	1408.16	14	100.58	63.9	<0.0001	significant
A	294.62	1	294.62	187.19	<0.0001	significant
B	51.05	1	51.05	32.43	<0.0001	significant
C	51.96	1	51.96	33.01	<0.0001	significant
D	234.44	1	234.44	148.95	<0.0001	significant
AB	0.3906	1	0.3906	0.2482	0.6261	
AC	4.41	1	4.41	2.8	0.1163	
AD	22.61	1	22.61	14.37	0.002	significant
BC	4.02	1	4.02	2.55	0.1323	
BD	0.0156	1	0.0156	0.0099	0.922	
CD	17.47	1	17.47	11.1	0.0049	significant
A^2^	455.46	1	455.46	289.37	<0.0001	significant
B^2^	328.21	1	328.21	208.52	<0.0001	significant
C^2^	199.85	1	199.85	126.97	<0.0001	significant
D^2^	45.36	1	45.36	28.82	<0.0001	significant
residual	22.04	14	1.57			
lost proposal	18.7	10	1.87	2.25	0.2263	insignificant
pure error	3.33	4	0.8329			
total deviation	1430.2	28				

**Table 6:** Analysis of the credibility of the model.

R^2^	R^2^_*adj*_	R^2^_*pre*_	CV (%)	Signal-to-Noise Ratio
0.9882	0.9763	0.9415	1.57	29.9645

**Table 7:** Experimental conditions for response surface model optimization.

Gas-Phase Flow Rate (mL/min)	Reaction Temperature (°C)	SO_3_/AO Molar Ratio	SO_3_ Volume Fraction (%)	Active Substance Content (%)
266	52	1.24	4.1	85.14

**Table 8:** The set of optimal solutions obtained by the decision.

Gas-Phase Flow Rate (mL/min)	Reaction Temperature (°C)	SO_3_/AO Molar Ratio	SO_3_ Volume Fraction (%)	Active Substance Content (%)	Power Consumption (kW)	Proximity to the Optimal Level
228	52.22	1.27	4	81.31	4.365	0.898
227	51.36	1.27	4.1	80.61	4.358	0.897
229	51.24	1.25	4	81.42	4.372	0.895
227	51.26	1.28	4.2	80.66	4.352	0.892
225	51.24	1.26	4	80.73	4.352	0.892

**Table 9:** Comparison between the dual objective solution and the response surface solution.

Type of Program	Gas-Phase Flow Rate (mL/min)	Reaction Temperature (°C)	SO_3_/AO Molar Ratio	SO_3_ Volume Fraction (%)	Active Substance Content (%)	Power Consumption (kW)
MOO	228	52.22	1.27	4	81.31	4.365
RSM	266	52	1.24	4.1	85.14	4.893

## Data Availability

The original contributions presented in this study are included in the article. Further inquiries can be directed to the corresponding author.
